# 

**Published:** 1898-02

**Authors:** 


### CONTENTS.

PROCEEDINGS.

Ohio State Dental Society ........ 51

COMMUNICATIONS.

Points ........ 70

SELECTIONS.

Health and Athletics ........ 72
A Peculiar Plant ........ 74
Meat-Eating ........ 74
Recuperating Effect of Sleep ........ 75
Digestion versus Drugs in the Treatment of Pulmonary Tuberculosis 76
Albert Edward, M. D. ........ 76
What Physicians Average per Year in New York ........ 76
Causes of Bad Taste and Odor in the Mouth ........ 77
Bread and Health ........ 77
Dr. Frank H. Hamilton's Pithy Points ........ 78
Fruit Eating ........ 78
Alcohol as a Beverage ........ 79
Don't Wet a Lead Pencil ........ 81
A Liberal Education ........ 82
Substitution ........ 82
Infection Through the Air ........ 83
A Simple Method of Preventing the Clouding of Mirrors ........ 84
Diagnosis of the Diseases of Children ........ 85
Doctors and Population ........ 85
Osteology ........ 87
Col. McKee's Bequest ........ 88
Quick Surgical Diagnoses ........ 88
Oil of Cinnamon ........ 88
Preservation of Hydrogen Peroxide ........ 89
The Continuity of Living Matter ........ 89
Mental Fatigue and Exercise ........ 89
Japanese Women and Suffrage ........ 89
Dirty Thermometers ........ 90
Buttermilk ........ 90
Buccelli on Effects of Tobacco ........ 90
Chicago Physicians Object to the Use of Cocaine ........ 90
X-Rays in Medicine and Surgery ........ 91
Be Good to Yourself ........ 91
Conversational Pitfalls ........ 91
How to Succeed ........ 92
Nausea from Chloroform Anesthesia ........ 92
A Curious Story in Relation to Hereditary Suicide ........ 92
Pure Air ........ 93
Vinegar After an Operation ........ 100

NOTICES.

Tenth Anniversary of the Odontographic Society of Chicago, Ills.... 97
Planting? or Implantation? Who First Gave This Operation to the Profession? ........ 97

EDITORIAL.

Ohio State Dental Society ........ 98
A Remarkable Man ........ 99

